# Review and new species of *Tiferonia* Darlington, 1962 (Carabidae, Abacetini)

**DOI:** 10.3897/zookeys.906.48255

**Published:** 2020-01-27

**Authors:** Kipling W. Will

**Affiliations:** 1 Essig Museum of Entomology, 1101 Valley Life Sciences Building, #4780, University of California, Berkeley, Berkeley, CA 94720- 4780, USA University of California Berkeley United States of America

**Keywords:** New Guinea, Africa, The Philippines, ground beetles, *
Holconotus
*, *
Melanchrous
*

## Abstract

Darlington described *Tiferonia* based on *T. parva* from New Guinea. In this review, *Tiferonia leytensis***sp. nov.** is described from Leyte Island, Philippines, *Tiferonia schoutedeni* (Straneo, 1943) **comb. nov.** is transferred from *Melanchrous* Andrewes, and inclusion of *Tiferonia brunnea* (Jedlička, 1935) in the genus is confirmed. Characteristics of *Tiferonia* and genera that have been proposed as closely related to *Tiferonia* are discussed and a unique character, the post-ocular sulcus, shared among species of *Tiferonia* and *Holconotus* is proposed as a synapomorphy for these two genera. A key to identify adults of the four species of *Tiferonia* is provided.

## Introduction

Darlington (1962) described the genus *Tiferonia* for two species from New Guinea and the Philippines but subsequently there have been no publications dealing with any additional specimens, species or taxonomic issues in the genus. Outside of checklists or catalogs, only a few papers have mentioned the genus as part of some larger study or peripheral to the principal paper topic. These typically only note the genus to distinguish it from of other genera, note its inclusion in abacetines or Loxandrini auctorum, or remark on its presence in the New Guinea fauna (Darlington 1962, 1971; Allen and Ball 1979; Allen 1982; Will and Park 2008; Will and Kavanaugh 2012). Specimens are quite rare in collections and there are no current efforts to collect in areas where species may exist using methods likely to obtain more material. This creates the very familiar problem of having only small numbers of specimens for study. During my recent investigations of various Abacetini genera and other Harpalinae that may have a relationship to abacetine taxa, including Darlington’s carabid specimens at the Museum of Comparative Zoology, it became apparent that there were several issues that need to be addressed in order to improve the state of the taxonomic understanding of *Tiferonia* with regard to species membership and possible phylogenetic relationships of this genus to other genera.

## Material and methods

Material examined. Specimens were examined from the following collections:

**ANIC**Australian National Insect Collection, Canberra;

**CMNH**Carnegie Museum of Natural History, Pittsburg, PA;

**CSCHM** J. Schmidt collection, Admannshagen, Germany;

**EMEC**Essig Museum of Entomology, Berkeley, CA;

**MCZ**Museum of Comparative Zoology, Cambridge, MA;

**NHM**The Natural History Museum, London.

Locality information for holotypes of the species described here is verbatim. Text as it appears on the labels is contained in quotation marks. The text for each label is delimited by double forward slash marks.

Images. Habitus photos of beetles were taken as image stacks that were aligned and assembled with Helicon Focus version 5.3 and image files were edited to enhance clarity using standard image editing software.

Dissection and measurements. Male genitalia were prepared using the same methods as Will (2002). Measurements were made using an ocular reticle. Standard body length (sbl) is the sum of the distance from the base of the labrum to just anterior of the occipital suture + the length of the pronotum along its midline + the length of the left elytron from basal margin where it meets the scutellum to the apex of the elytron. The width of the elytra is the widest point viewed dorsally. The ocular ratio is the width over the eyes divided by the width between the eyes measured at the level of the anterior supraocular setae, viewed dorsally. Measurements and ratios are given for the type specimen and then a range of all specimens measured is given in brackets.

## Taxonomic treatment

### Abacetini Chaudoir, 1873

#### Tiferonia

Taxon classificationAnimaliaColeopteraCarabidae

Darlington, 1962

8ACFA0F6-6419-5C37-A03F-0A9411048D48

Tiferonia Darlington, 1962: 560.

##### Type species.

*Tiferonia parva* Darlington, 1962: 562, by original designation.

##### Generic diagnosis.

**Figure 1.**
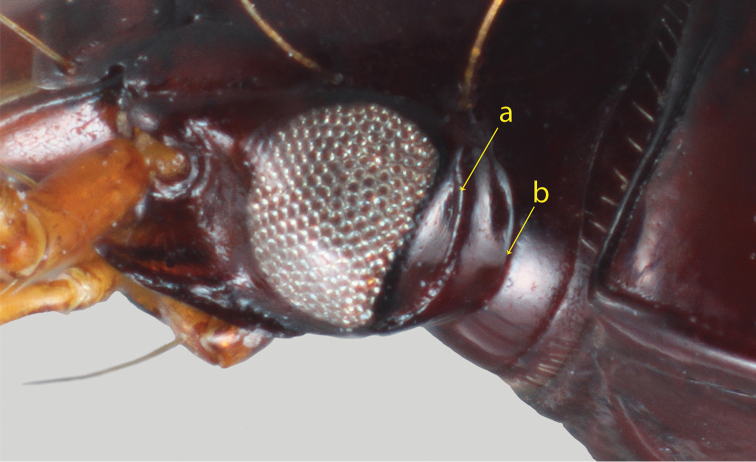
*Tiferonia leytensis* sp. nov. head, left lateral view. Arrows indicate **a** post-ocular sulcus and **b** posterior edge of the orbit.

With a combination of typical abacetine characters such as clearly defined frontal impressions on the head; deeply impressed, linear basolateral pronotal impressions; no angular base of stria 1 on elytra; setose puncture at the base of elytral stria 2; well-developed elytral plica; metacoxal sulcus sinuate; abdominal ventrites without transverse sulci; ostium of aedeagus dorsal; and aedeagus left side dorsal in repose. Recognizable from other abacetine genera that share the character states listed above by the combination of deep post-ocular sulcus (Fig. 1), smooth elytral margins, and lack of elytral discal setae.

##### Genus characteristics.

**Figures 2, 3.**
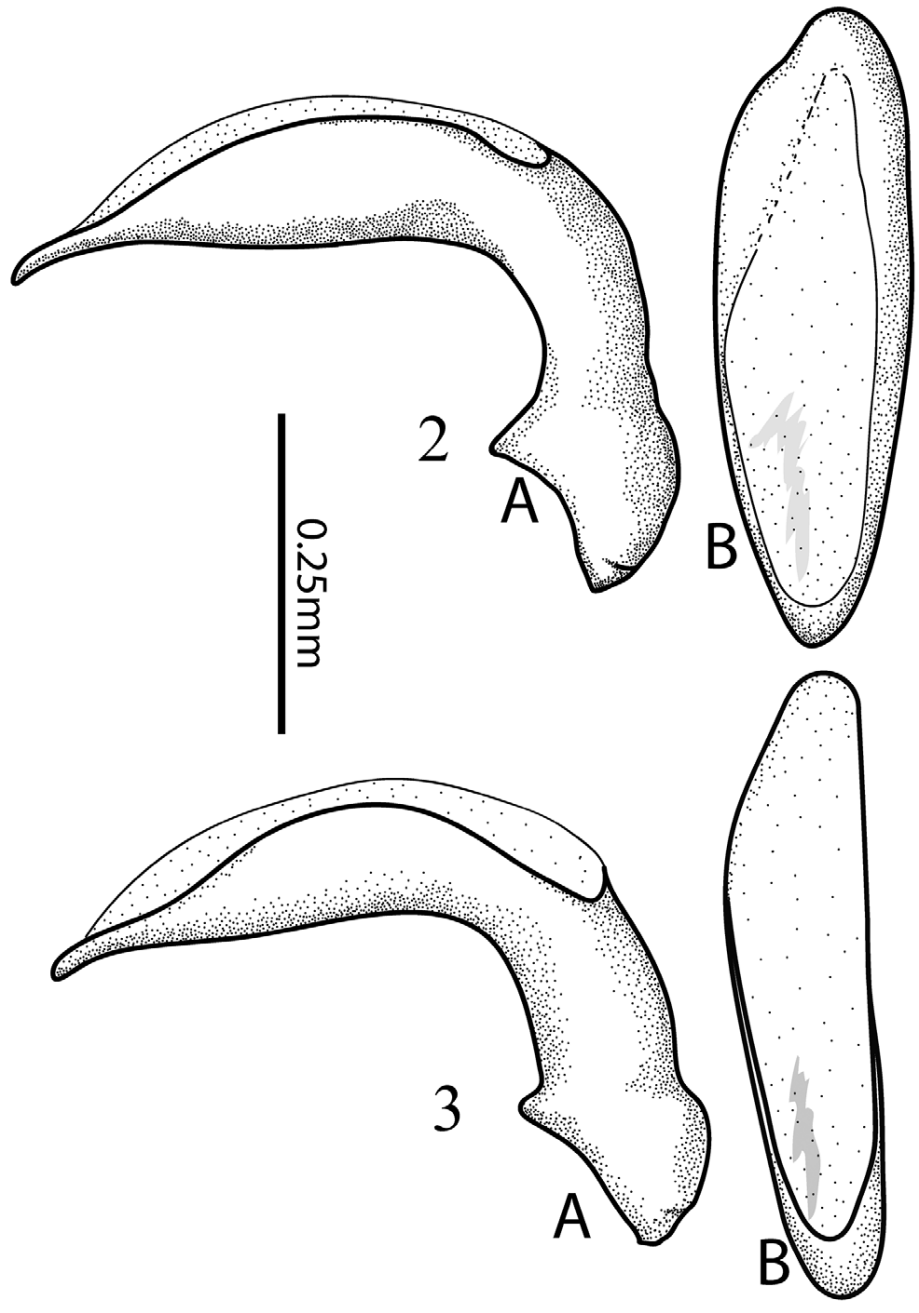
*Tiferonia* species aedeagi, right lateral view (**A**) and dorsal view (**B**). **2***Tiferonia leytensis* sp. nov. **3***Tiferonia parva*.

Small size beetles (3.8–4.3 mm), castaneous or darker, nearly piceous colored, parallel sided, somewhat convex body form; apical segment of labial palpi elongate and fusiform. Mentum narrow triangular, shallowly emarginate; epilobes long and narrow, not prominent; median tooth prominent and entire, not reaching tips of lobes, mentum paramedial pits absent; paraglossae short, glabrous; submentum narrow, posteriorly sculpted; antennae of moderate length, somewhat thickly filiform, three basal segments glabrous except for apical ring of setae; postocular orbits moderately pronounced, with deep post-ocular sulcus (Fig. 1). Elytra free, lateral margin smooth; border entire across base; parascutellar stria present, joined to stria 1; 13–15 umbilicate setae in stria 8; hind wings fully developed; humeri obtusely angled with very small, usually sharp denticle; anterior tarsi of male with three basal segments narrowly dilated and squamose beneath. Aedeagus (Fig. 2) with orifice on dorsum; parameres conchoid, the right smaller than the left.

#### Tiferonia parva

Taxon classificationAnimaliaColeopteraCarabidae

Darlington, 1962

B65E42A9-5BAD-5498-8A9D-2C7A8E7E5A91

Figs 3 , 5 , 6

##### Material examined.

***Holotype***: New Guinea • ♂, (M.C.Z. No. 30,231). //“Aitape Brit. N.G. Aug 1944”// “det. Darlington at B.M. 1947-48 Notes p.”// “Genus.? Det. Darlington”// “Meas. ♂”// “gen. Melanchrous Andr. det. S.L.Straneo 1953”// “Tiferonia parva Darl.”// “M.C.Z. Type 30231”//.

***Paratypes***: New Guinea • 1♂, 1♀, same data as holotype • 1♂, // “vic. Hollandia Dutch N.G. July-Sept 1944 Darlington”// [ANIC].

##### Diagnosis.

Very similar to *T. leytensis* but distinct from that species by the form of the pronotum, which is broad and straight onto the base and the form of the male genitalia (Figs 2, 3).

#### Tiferonia brunnea

Taxon classificationAnimaliaColeopteraCarabidae

(Jedlička, 1935)

768D16A4-6F49-5631-A984-6F8F6CEB7D3A

Fig. 9

Fouquetius brunneus Jedlička, 1935: 108.
Holconotus brunneus : Lorenz 2005: 259.
Tiferonia brunneus : Darlington 1962: 561.

##### Material examined.

***Holotype***: Philippines • ♂, // “Mt. Makiling, Luzon Baker”// “H.E.Andrewes Coll. BM 1945-97.”// [red label, black border, printed] // “TYPUS”// //[pink label, handwritten and print] “Fouquetius brunneus type sp. nov. DET H. ANDREWES”// [NHM].

##### Type locality.

Estimated to be centered on 14.1346N, 12.1955E, south east of Calamba.

##### Diagnosis.

The single pair of supraorbital setae distinguishes this species from all other species of *Tiferonia*.

#### Tiferonia schoutedeni

Taxon classificationAnimaliaColeopteraCarabidae

(Straneo, 1943) comb. nov.

34A465E2-0048-5754-9298-AAD3C7566C7D

Fig. 8

Patellus schoutedeni Straneo, 1943: 5.
Melanchrous schoutedeni : Straneo 1962: 54; Lorenz 2005: 328.

##### Notes.

According to Straneo (1943) the type was intended to be deposited in Tervuren. However, the specimen was not located (S. Hanot, Musee Royal de l’Afrique Centrale, Tervuren, Belgium, in litt.). Straneo stated that the specimen is labelled “Congo Belge, Eala (17-I-1921, Dr. H. Schouteden).”

##### Material examined.

Central African Republic • 1♀, //“R[epublic] C[entral] A[frica], P[ark]. N[ational]. [Dzanga-]Ndoki, Camp1 02 28 51.0N 016 13 04.5E, 9–11.II.2012, piége UV canopée 35m, Exp. Sangha 2012, P. Moretto leg. -70-”// [CSCHM] • ♂ //“Bot. N°69 Humus dans résidu forestier”// “I[nstitut pour la]. R[echerche]. S[cientifique en]. A[frique]. C[entrale]. –Mus. Congo Kwango: terr, de feshi, rive dr. Kwenge III-1959 B. 69 Mme J. Leleup”// “*Melanchrous schoutedeni* S.L. Straneo det. 1960”//[CMNH].

##### Diagnosis.

Having only the first three elytral intervals impressed and a relatively large eyes (gena is only half the width of antennomere 1) distinguishes this species from all other *Tiferonia*.

#### Tiferonia leytensis sp. nov.

Taxon classificationAnimaliaColeopteraCarabidae

4D3EDE20-AB6F-57C3-B79E-98D70B15486C

http://zoobank.org/939FDB9B-0561-473C-921D-D9AD409E87BF

Figs 1 , 2 , 4 , 7

##### Material examined.

***Holotype***: Philippines • ♂, // “Plains of NE Leyte Is.,P.I. Nov ’44-Jan’45 Darlington”// “MCZ Holotype 36215”// [deposited MCZ].

***Paratypes***: Philippines • 1♂, 1 ♀, same data as holotype [MCZ].

##### Type locality.

As listed on locality label, type locality is estimated to be roughly centered on 11.221N, 124.828E.

##### Diagnosis.

**Figures 4, 5.**
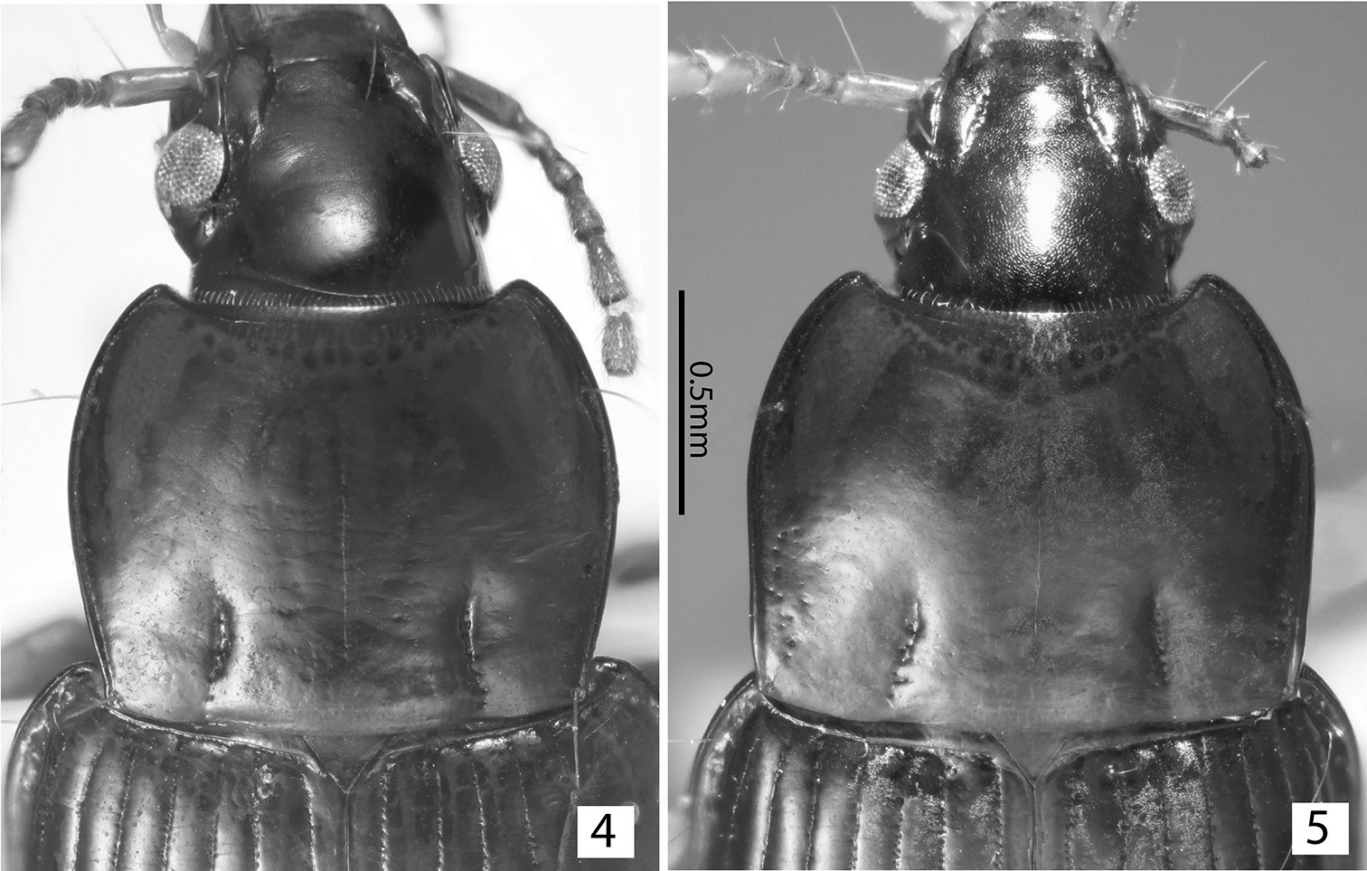
*Tiferonia* species pronota. **4***Tiferonia leytensis* sp. nov. **5***Tiferonia parva*.

The combination of two pairs of supraorbital setae, all elytral striae impressed, and the pronotum (Fig. 4) basally narrowed with slightly sinuate lateral margins separates this species from all other species of *Tiferonia*.

##### Description.

***Size.*** Overall length (sbl) 3.96 mm [3.96–4.21 mm]; greatest width over elytra 1.65 mm [1.62–1.79 mm]. ***Color.*** Dorsal and ventral surfaces piceous to dark rufous, head slightly darker, elytral interval 1 and apical fourth of elytra paler; legs, mouthparts, and antennae pale brunneous, tibiae darker rufous. ***Luster.*** Dorsally and ventrally distinctly shiny. Iridescence not evident on head, spectral iridescence slightly apparent on pronotum, distinctly evident on elytra, slightly apparent on ventral surface of body. ***Head.*** Dorsal microsculpture evident nearly isodiametric mesh. Clypeal-ocular impressions clearly impressed, narrow, divergent, and extended to anterior supraocular seta. Ocular ratio 1.61 [1.55–1.58]. Eyes moderate size, prominent, with large posterior orbital area; gena slightly narrower than width of antennomere 1. Labrum with anterior margin straight. Mentum median tooth entire, triangular. Antennae, overall length moderately long, antennomeres 10–11 surpassing pronotal base. ***Thorax.*** Pronotum dorsally impunctate, microsculpture not evident at 50× magnification in anterior half, irregular, very transverse mesh slightly evident near base; widest at middle, lateral margins with short, slight sinuation near hind angles; lateral marginal bead uniformly evident and narrow throughout; basal margin smooth, not beaded; anterior angles moderately produced; anterior submarginal sulcus broadly interrupted medially; hind angles right angled, denticulate; basal impressions linear, deeply impressed, slightly crenulate, shallowly reaching basal margin; seta at hind angle touching marginal bead. Elytra parallel sided; plica large and externally visible. Elytral striae well impressed, deeply, densely crenulate-punctate; intervals nearly flat, slightly convex basally. Elytral microsculpture scarcely evident as very transversely stretched sculpticells. Prosternal process rounded, margin not marked with bead; prosterna and proepisterna smooth. Mesosterna with few, coarse punctures. Metasternum laterally and metepisternum with shallow, coarse punctures. ***Abdomen.*** Abdominal ventrites irregularly, coarsely punctate laterally, impunctate medially. ***Male genitalia*** (Fig. 2), ostium dorsal, endophallus with light spine field in left apical position in repose. ***Female ovipositor*** moderately long, slightly curved, two large ensiform setae, one dorsal one ventral, two long nematoform setae in well-developed groove.

**Figures 6–9.**
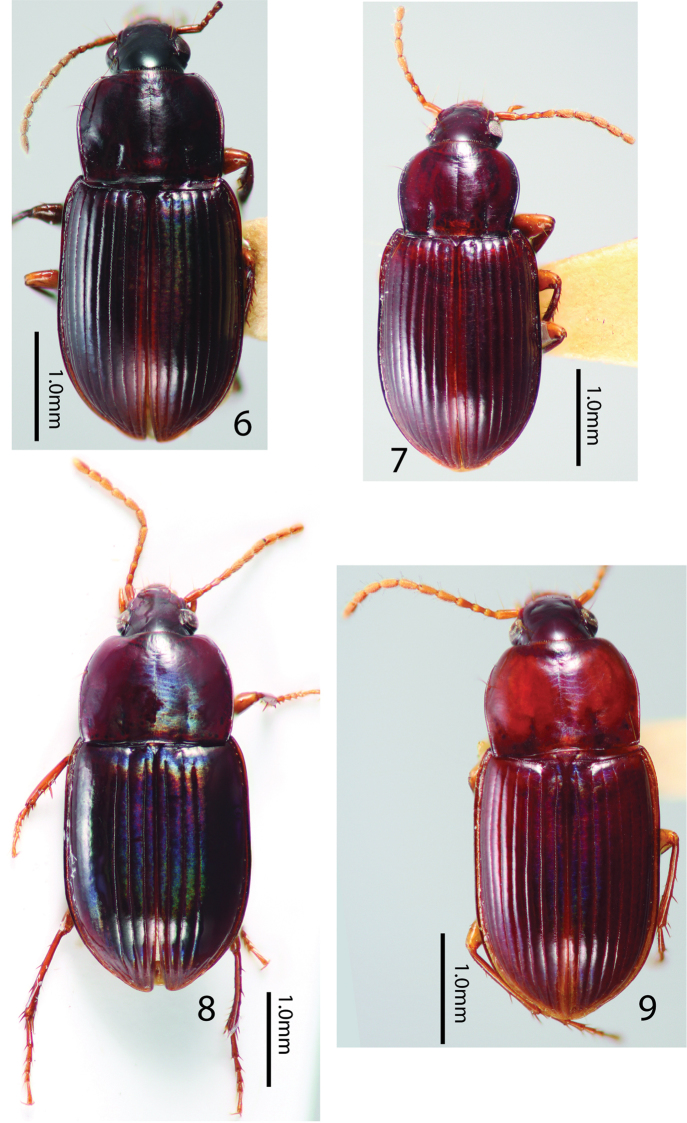
*Tiferonia* species dorsal habitus. **6***Tiferonia parva*, male paratype **7***Tiferonia leytensis* male holotype **8***Tiferonia schoutedeni***9***Tiferonia brunnea*, male holotype.

##### Etymology.

The specific epithet *leytensis* is based on the type locality and is treated as an adjective.

##### Notes.

In his discussion of the species of *Tiferonia*, Darlington (1962: 561) states that *T. brunnea* from the Philippines is distinguished by having only a single pair of supraorbital setae and then states that he has a series of that species from Leyte Island. However, this appears to be an error. Among Darlington’s specimens at the MCZ he has a series from Leyte Island, but they all have two pairs of supraorbital setae and are otherwise distinctly different from *T. brunnea*. These specimens comprise the holotype and paratypes of *T. leytensis*.

### Possible evolutionary relationships of *Tiferonia*

Darlington (1962) noted that *Tiferonia* was “superficially similar to *Melanchrous.” Melanchrous* was treated as a member of Oodini by Chaudoir (1883) but then moved to Melanchitonini by Straneo (1962) and has remained in that tribe in recent catalogs (Lorenz 2005a, b; Bousquet 2012). The holotype of *T. parva* bears a determination label written by Straneo from 1953 with “gen. *Melanchrous* Andr.,” which is likely what suggested this comparison to Darlington. He then points out that *Melanchrous* from southeast Asia and the Malay Archipelago have protarsomeres with densely pubescent pads ventrally, similar to what is found in some melanchitonines and oodines, not biseriately squamulose as in *Tiferonia* and other abacetines. I have examined types or confidently identified specimens of all *Melanchrous* species except for one of the three the African species, *Melanchrous celisi* Straneo, 1962. All examined *Melanchrous* species differ from *T. schoutedeni* by having protarsomeres with densely pubescent pads ventrally, not squamulosely biseriate. Additionally, no species of *Melanchrous* has the post-ocular sulcus found in *Tiferonia* and *Holconotus* (Fig. 1). The type specimen of *M. celisi* could not be located (S. Hanot in litt.) and I have not seen any specimens that agree with Straneo’s description of the species. Straneo described *M. celisi* in comparison to *T. schoutedeni*, to which it is similar in having a reduced number of impressed striae, but no character states were reported that can verify or refute its placement in *Melanchrous*.

*Tiferonia* and *Holconotus* are both abacetine genera that appear to be close relatives. Darlington (1962) included Jedlička’s *brunneus* in *Tiferonia* while noting that *Holconotus* (= *Fouquetius*) has “dentate humeri and serrate elytral margins,” which he states *Tiferonia* does not. While it is correct that all *Holconotus* have these states, it is not the case that the humeral tooth is lacking in *Tiferonia*. The tooth is slightly smaller and, in some cases, more rounded than typically observed in *Holconotus*, but always present. The humeri in *Melanchrous* (see above) is fully rounded, with no suggestion of a tooth. The presence of the serrate elytral margin is likely a synapomorphy for *Holconotus* species, excluding *Tiferonia*. The shared post-ocular sulcus appears to be a good synapomorphy for a sister-group relationship for *Tiferonia* and *Holconotus*. No other genera of Abacetini, and to my knowledge no other carabids, have the post-ocular sulcus as in these two genera.

### Key to adults

**Table:** 

1	Elytron with eight striae impressed from the apex to or nearly to the base	**2**
–	Elytron with only the first three striae impressed from the apex to, or nearly to the base (Fig. 8). Africa	***Tiferonia schoutedeni* (Straneo, 1943)**
2	Two pairs of supraorbital setae	**3**
–	One pair of supraorbital setae. The Philippines	***Tiferonia brunnea* (Jedlička, 1935)**
3	Pronotum lateral margins slightly sinuate in the basal third, base notably narrower than elytra (Fig. 4). Male aedeagus wide and sharply narrowing at tip in ventral view (Fig. 2). The Philippines	***Tiferonia leytensis* sp. nov.**
–	Pronotum lateral margins nearly straight in the basal third, base nearly as wide as elytra (Fig. 5). Male aedeagus narrow and blunt at tip in ventral view (Fig. 3). New Guinea	***Tiferonia parva* Darlington, 1962**

## Supplementary Material

XML Treatment for Tiferonia

XML Treatment for Tiferonia parva

XML Treatment for Tiferonia brunnea

XML Treatment for Tiferonia schoutedeni

XML Treatment for Tiferonia leytensis
